# Unravelling the palaeobiogeographical history of the living fossil genus *Rehderodendron* (Styracaceae) with fossil and extant pollen and fruit data

**DOI:** 10.1186/s12862-022-02097-4

**Published:** 2022-12-15

**Authors:** Christa-Charlotte Hofmann, Wan-Yi Zhao

**Affiliations:** 1 Department of Palaeontology, University Vienna, Josef Holaubek-Platz 2, 1090 Vienna, Austria; 2 State Key Laboratory and Guangdong Key Laboratory of Plant Resources, Sun Yat-sen University, Guangzhou, 510275 China

**Keywords:** Cenozoic palaeobiogeography, Fossil pollen, Fossil fruits, *Rehderodendron*

## Abstract

**Background:**

The relict genus *Rehderodendron* (Styracaceae), the species of which are restricted to mostly warm temperate to tropical climate in East Asia today, is known from fossil fruits and pollen in Europe during warmer periods from the lower Eocene to Pliocene. To infer which extant species are most closely related to the fossils, new data of pollen and fruit morphologiesy of six extant species, and additional new data of fossil pollen and previously described fossil fruits of *Rehderodendron*, are compared.

**Results:**

Both fossil pollen and fruits resemble a morphological mixture of the extant species *R.* *indochinense,* *R.* *kwantungense,* *R.* *macrocarpum,* and *R.* *microcarpum,* thus implying that these extant taxa and the fossil European taxa represent an old Eurasian lineage, whereas the pollen and fruit morphology of the extant *R.* *kweichowense* and *R.* *truongsonense* differ considerably from the fossils and other extant species investigated, and are considered to have evolved independently.

**Conclusions:**

The palaeobiogeographical history of *Rehderodendron* reveals that its fossil members of the European lineage were most prominent during climatic optima such as the Palaeocene–Eocene Thermal Maximum (PETM), Early Eocene Climate Optimum (EECO) and Middle Miocene Thermal Maximum (MMTM). However, when during the Pliocene the climate changed to colder and less humid conditions, the genus went extinct in Europe but migrated eastwards, most likely in two dispersal events along the Tethys Sea prior to extinction. One of the former most westerly stepping stones is suggested by the refugial occurrence of *R.* *microcarpum* in the southeastern Himalaya, whereas *R.* *macrocarpum* and *R.* *kwangtungense*, the taxa distributed more to the east, might have migrated eastwards already before the Miocene.

## Introduction

The current flora of eastern Asia and southeastern Europe is assumed to be a relict of an ancient Cenozoic flora that thrived along the warm and humid northern margin of the Tethys Sea. The palaeoflora in Eurasia was characterized by a high degree of uniformity [e.g., [1–5] and underwent changes from the Palaeocene to Miocene and Pliocene in response to climatic changes [6, 7] and the closing of the Tethys. After the Pliocene, the thermophilic elements disappeared completely from Europe either prior or during Quaternary glaciation, but many of them still persist in eastern Asia [5]. Manchester et al. [3] considered that some of the extant genera exhibit morphological stasis and therefore can be considered “living fossils” traced back to early Cenozoic times. *Rehderodendron* appears to be one such fossil, because it was reduced from a widespread geographic distribution during the Cenozoic to only eastern Asia in modern times in response to environmental and climatic changes. Comparable examples are *Carya* Nuttall (Juglandaceae), which was more diverse in Europe than in North America and Asia during the Neogene, but now has disappeared completely from the modern European flora [8, 9], and *Styrax* Linnaeus (Styracaceae), whose evolutionary history can be traced the through the Cenozoic relictual flora in Europe [10] but today is only present with one species in south east Europe, whereas although it is diverse today in Asia and the Americas.

**Fig. 1:**
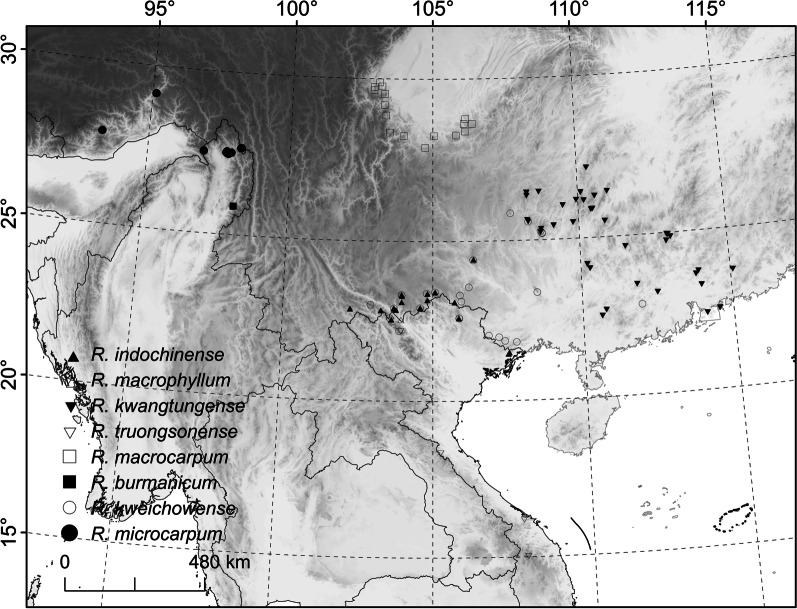
Map of Asia showing geographic distribution distribution of the extant species of *Rehderodendron*

The Styracaceae family comprises 12 genera and ca. 160–180 woody plant species occurring in warm temperate to tropical climates in the Americas, south Europe, east and south east Asia and Malesia [11, 12]. The family forms a well supported clade [10, 12], and most of the genera are monotypic or oligotypic with limited geographic distribution, except *Styrax*, the largest genus [10, 13]. *Rehderodendron* Hu comprises 6–8 species that occur as trees in China, Vietnam and, Myanmar (Fig. 1; Table 1; [10, 11, 14–17]). Most of the *Rehderodendron* species are deciduous trees and flower before their leaves develop (*R.* *burmanicum* (W.W. Sm. & Farrer) W.Y. Zhao, P.W. Fritsch & W.B. Liao*,* *R.* *indochinense* H.L. Li*,* *R.* *kwangtungense* Chun, *R.* *kweichowense* Hu, *R.* *macrocarpum *Hu, *R.* *microcarpum* K.M. Feng ex T.L. Ming) and grow in montane evergreen and mixed deciduous broadleaved forests. Two species (*R.* *truongsonense* P.W. Fritsch, W.B. Liao & W.Y. Zhao and *R.* *macrophyllum* (C.W. Wu & K.M. Feng) W.Y. Zhao, P.W. Fritsch & W.B. Liao; [17]) are considered evergreen and occur in ravine seasonal rain forests or broadleaved montane evergreen forests (Table 1). The fruit of *Rehderodendron* is distinguished from those of the other Styracaceae genera by its large size and cylindrical shape, harbouring an endocarp with many irregular rays intruding into the mesocarp [15, 17].

**Table 1:** List of extant species investigated (p = pollen; f = fruits), their collection sites, general occurrences and climate according to Köppen-Geiger [44, 45] and Köppen-Trewartha [46]

Species pollen and fruits (p, f)	Collector	Voucher	Location	Date	GPS	General occurrence	Vegetation type	Koöppen-Geiger	Köppen-Trewartha
*Rehderodendron kweichowense* Hu (p & f)	W.Y. Zhao, F. Ye & Y.Z. Chen	ZWY-1349	China: Yunnan province, Pingbian County, Daweishan National Natural Reserve	9.04.2019	22°54′N 103°42′E	N Vietnam: Chapa, Cao Bang, Lao Cai; China: Guangxi, Guangdong, Guizhou, Yunnan	Montane monsoon forest	Cwa (Cwb) Cfa	Cw Cf
*Rehderodendron kwangtungense* Chun (p & f)	W.Y. Zhao, F. Ye & C. Liao	ZWY-1681	China: Guangdong province, Ruyuan County, Nanling National Natural Reserve	11.04.2020	24°52′N 113°5′E	China: Guangxi, Guangdong, Guizhou, Hongkong, Hunan	Montane evergreen and deciduous broadleaved mixed forest	Cfa Cwa	Cf Cw
*Rehderodendron macrocarpum* Hu (p & f)	W.Y. Zhao, F. Ye & Y.Z. Chen	ZWY-1500	China: Sichuan province, Leibo County, Shanlenggang Township, Qinlangdang village	21.04.2019	28°20′N 103°28′E	China: Chongqing, Guizhou, Sichuan, Yunnan	Montane deciduous broadleaved forest	Cwb Cwa Cfa	Cw Cf
*Rehderodendron microcarpum* K.M.Feng ex T.L.Ming (p & f)	W.Y. Zhao & F. Ye	ZWY-1389	China: Yunnan province, Gongshan County, Dulongjiang Township, Qinlangdang village	13.04.2019	27°41′N 98°16′E	China: Yunnan, Xizang; N-Myanmar: Kachin State	Montane monsoon forest or mountain evergreen and deciduous broadleaved mixed forest	Cwa (Cwb)	Cw
*Rehderodendron truongsonense* P.W. Fritsch, W.B. Liao, & W.Y. Zhao (p & f)	U. Swenson, D.V. Truong, H.M. Quyen, & S. Razafimandimbison	2131	Vietnam: Ha Tinh province, Vu Quang National Park, S of Khe Che Forest Station	9.10.2018	18°22′30"N 105°18′42"E	N–C Vietnam: Chapa, Ha Tinh province, Da Nang, Kon Tum, Quang Nam	Ravine seasonal rain forests or montane evergreen broadleaved forest	Am Cwa (Cwb)	Aw Cw
*Rehderodendron indochinense* H.L. Li (p & f)	W.Y. Zhao, F. Ye & C. Liao	ZWY-1559	China: Yunnan Province, Malipo County, Xiajinchang Township	28.03.2020	23°10′N 104°49′N	N Vietnam: Caobang, Chapa, Ha Coi; China: Guangxi, Yunnan	Montane monsoon forest (evergreen and deciduous broadleaved mixed forest)	Cwa (Cwb)	Cw

The earliest recognizable fossil occurrences of *Rehderodendon* are fruits of *Rehderodendron* *stonei* (Reid & Chandler) Mai from the lower Eocene of England [18, 19] and from the middle Eocene of France [20]. Other Styracaceae fossils are fruits of *Styrax* spp. from the upper Eocene of England [21] and pollen of *Styrax* from the lower Eocene of Austria [22]. However, because of their characteristic fruit morphology, fossil *Rehderodendron* fruits have been recognized subsequently from other fossil localities in Europe ranging from Miocene to Pliocene in age. Leaf morphology is of limited utility in distinguishing among Styracaceae genera. No fossil leaves of *Rehderodendron* have been described.

The overall pollen morphology of *Rehderodendron* is typical for Styracaceae in general, but until now only a few meagre descriptions and images of extant *Rehderodendron* pollen exist, mainly in a broader context circumscribing the pollen morphology of the family Styracaceae [23, 24]. The pollen often are depicted with scanning electron microscopy (SEM), light microscopy (LM) or transmission electron microscopy (TEM); however, the information from these images is often limited, mainly because of low magnification or insufficient printing techniques. Despite the fact that the endemic distribution of *Rehderodendron* today is mainly in China, Vietnam and Myanmar, and the fact that fossil *Rehderodendron* diaspores have been recorded only from Europe since the lower Eocene to the Pliocene [3, 18, 25–28], with few exceptions fossil pollen of *Rehderodendron* is pretty scarce in the literature [e.g. 29, 30]. Here we present original LM, SEM images and descriptions of pollen from six extant species of *Rehderodendron* (*R.* *indochinense*, *R.* *kwangtungense,* *R.* *kweichowense,* *R.* *macrocarpum,* *R.* *microcarpum,* and *R.* *truongsonense*) and compare them with LM and SEM images of three fossil *Rehderodendron* pollen from lower Eocene and middle Miocene strata of England, Austria and Germany. Additionally we provide new images and descriptions of extant fruit morphology of *Rehderodendron* and all these results are used to reconstruct the palaeobiogeographical history and evolution of this living fossil.

## Methods and material

Flower and fruit material of *Rehderodendron* *kwangtungense* *R.* *kweichowense*, *R.* *macrocarpum*, *R* *microcarpum* and *R.* *indochinense* from China was collected by Zhao W.Y. and his Chinese and International colleagues in 2018 and 2020, and identified by him. *Rehderodendron* *truongsonense* from Vietnam was collected by U. Swenson and colleagues in October 2018 (Table 1). All plants are housed in Sun Yat-sen University, State Key Laboratory and Guangdong Key Laboratory of Plant Resources (Guangzhou China).

Anther material of *Rehderodendron* species was soaked in a drop of acetolysis mixture (9:1 acetic acid anhydride: concentrated sulphuric acid) on a glass slide under a binocular and manipulated with a needle to release the pollen from the anthers. The anthers in the acetolysis mixture were repeatedly heated for several seconds over a candle flame to colour the pollen wall and extrude the cell contents. Then the pollen grains were fished out with a micro-manipulator (eyebrow hair mounted on a needle) and transferred to a clean drop of glycerol for LM photography together with a micrometer (Nikon). After photography, the pollen grains were transferred to SEM stubs with a micro-manipulator into minute drops of alcohol to wash off the remaining glycerol, then the stubs were sputter-coated with gold (BIO-RAD) under argon atmosphere and investigated in high vacuum with a FEI Inspect S 500 scanning electron microscope.

The fossil pollen grains were recovered from sediment samples covering the Palaeocene–Eocene-Thermal Maximum (PETM) from England and the Middle-Miocene-Thermal Maximum (MMTM) from Austria and Germany [22, 29, 31] by treating the sediments with HF and HCL with subsequent acetolysis [e.g., 32, 33]. The remaining extracts were mixed with glycerol and smeared onto a glass slides. The manipulation, photography and SEM investigation of fossil pollen followed the same procedures as for the extant pollen. SEM stubs of the fossil pollen are housed in the Department of Palaeontology, University of Vienna under IPUW number 7838a, 7841, 7843. The morphological characters of fossil *Rehderodendron* fruits were modeled on descriptions in previous studies [18, 20, 26–28].

## Results

**Fig. 2:**
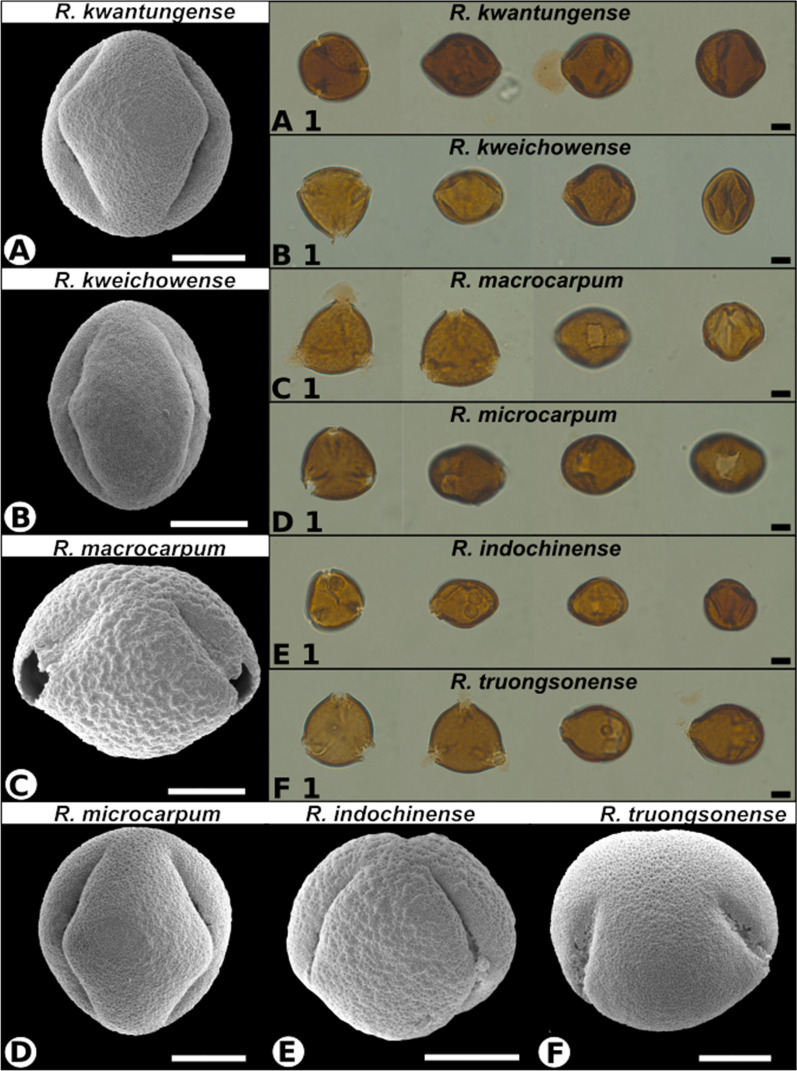
LM and SEM overview images of extant *Rehderodendron* pollen. Scale bar in the LM images 10 µm, scale bar in the SEM overview images 10 µm

**Fig. 3:**
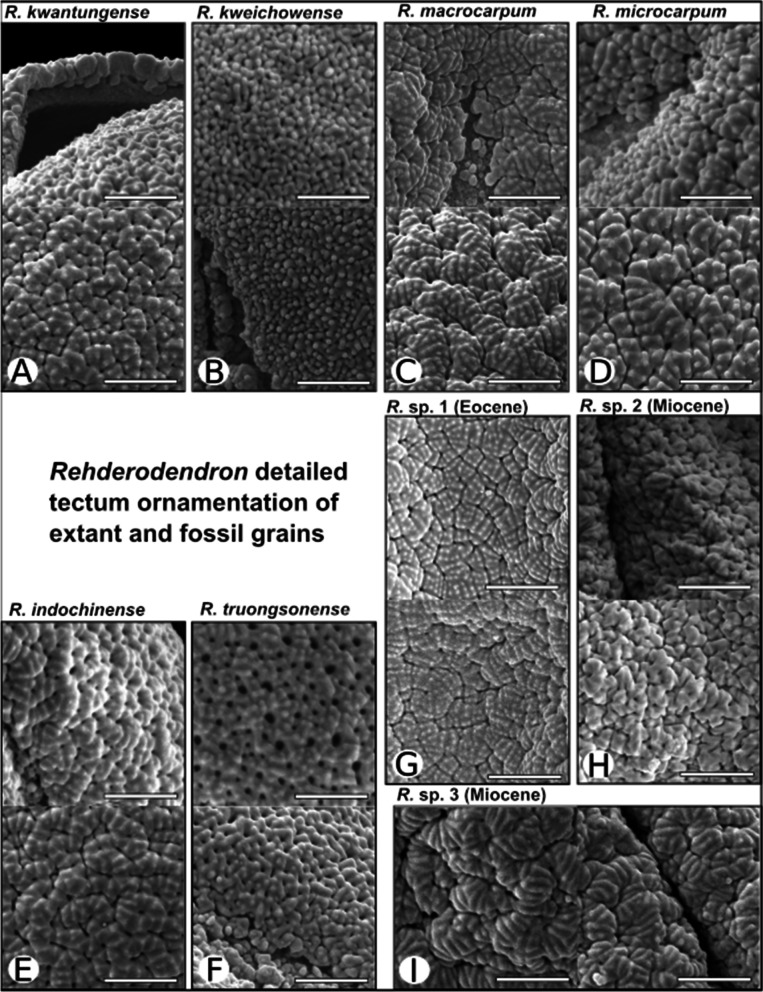
SEM detail images of extant and fossil *Rehderodendron* pollen: Scale bar = 2 µm

Pollen grains of six extant *Rehderodendron* species were photographed under LM and SEM and their sizes measured (Table 2, Figs. 2, 3). Depending on the length of heating during the acetolysation process, the pollen changed shape from originally suboblate (unacetolysed state) to subspheroidal and then to more subprolate (fully acetolysed state; Fig. 2). Pollen measurements displayed considerable differences: LM photographs of pollen in glycerol with a micrometer scale yielded larger sizes than the measurements of pollen photographed with SEM (more-or-less desiccated state of pollen after being washed in alcohol, partly desiccated and sputter-coated under argon atmosphere; Table 2, Fig. 2). The measurements of the width and height of the endopori was only possible under LM. However, all measured sizes fall within the size ranges of previously measured *Rehderodendron* pollen in [23 page 87: *R.* *kwangtungense,* *R.* *kweichowense* and *R.* *indochinense*, the last = is *R.* *macrocarpum* according to the junior author] and [24 Table 1: *R.* *macrocarpum*]. The same is true for the fossil *Rehderodendron* pollen (see descriptions below).

**Table 2:** Pollen size measurements of extant species

*Rehderodendron* *species*	N	Equatorial axes LM min–max	Equatorial axes SEM min–max	Polar axes LM min–max	Polar axes SEM min–max	Porus width LM min–max	Porus height LM min–max
*R.* *indochinense*	10	30.9–38.2	23.1–37.5	26.9–29.1	23.5–27.1	5.4–6.7	5.4–9.1
*R.* *kwantungense*	10	34.5–42.7	26.2–31.2	32.4–38.7	27.8–29.0	5.4–7.4	7.2–11.8
*R.* *kweichowense*	19	17.8–39.6	20.9–29.7	27.3–34.9	25.0–27.2	2.7–9.8	3.2–8.4
*R.* *macrocarpum*	13	32.0–44.2	24.3–33.6	28.2–34.2	24.3–26.3	6.4–10.4	4.9–11.8
*R.* *microcarpum*	9	37.3–42.4	26.6–31.2	31.8–32.7	26.2–28.8	5.9–9.1	10.4–11.9
*R.* *truongsonense*	12	36.4–40.0	28.3–32.1	30.5–32.7	26.1–28.2	4.3–7.4	6.3–12.7

### Description of extant pollen

Ericales Dumortier

Styracaceae Dumortier

*Rehderodendron* Hu

#### *R. kwangtungense* Chun (Figs. 2A, A1, 3A)

Pollen grains tricolporate (rarely tetracolporate), spheroidal to subprolate, triangular to circular in polar view and angular, elliptical to subcircular in equatorial view; measurements under LM: polar axes 32.4–38.7 µm, equatorial axes 34.5–42.7 µm; under SEM: polar axes 27.8–29.0 µm, equatorial axes 26.2–31.2 µm; endoporus rectangular to quadrangular: 7.2–11.8 µm × 5.4–7.4 µm (Figs. 1, 2). Tectum: tectate, perforate, shallowly fossulate, and faintly rugulate with rugulae bordered by faint fossulae or perforations (comparable to *R.* *indochinense*, but less pronounced), towards the colpi more micro-verrucate; ectexine in polar areas and mesocolpi regularly ornamented with supratectal blunt micro-echini (or micro-gemmae), colpus membrane, when visible, micro-verrucate; pollen wall 1.2–1.3 µm thick with sexine (0.7–0.8 µm) thicker than nexine (0.4–0.5 µm), endexine columellar to granular (columellae max. 0.3 µm high).

#### *R. kweichowense* Hu (Figs. 2B, B1, 3B)

Pollen grains tricolporate, suboblate, subspheroidal to prolate, triangular to circular in polar view and angular, elliptical to subcircular in equatorial view (Figs. 1, 2); measurements under LM: polar axes 27.3–34.9 µm, equatorial axes 17.8–39.6 µm; under SEM: polar axes 25.0–27.2 µm, equatorial axes 20.9–29.7 µm; endoporus rectangular to quadrangular 3.2–8.4 µm × 2.7–9.8 µm. Tectum: tectate, micro-verrucate to occasionally micro-rugulate, perforate, diameter of micro-verrucae ca. 0.2 µm, several micro-verrucae and micro-rugulae locally fused to produce areolae of 0.5–0.8 µm in diameter; colpus membrane, when visible, micro-verrucate; pollen wall thickness 1.1–1.3 µm with sexine thicker than nexine.

#### *R. macrocarpum* Hu (Figs. 2C, C1, 3C)

Pollen grain tricolporate, suboblate, subspheroidal, triangular to circular in polar view and angular, elliptical to subcircular in equatorial view (Figs. 1, 2); measurements under LM: polar axes 28.2–34.2 µm, equatorial axes 32–44.2 µm; under SEM: polar axes 24.3–26.3 µm, equatorial axes 24.3–33.6 µm; endoporus shape rectangular to quadrangular 4.9–11.8 µm × 6.4–10.4 µm. Tectum: tectate, perforate fossulate, fossulae border ± elongated, occasionally curved, angular rugulae (or areolae) of 0.3–0.8 µm width and 1.2–2.8 µm length; rugulae flat, with regularly spaced rows composed of occasionally fused supratectal micro-echini (or micro-gemmae) arranged perpendicularly to rugulae; colpus membrane, when visible micro-verrucate; pollen wall thickness 1.4–1.7 µm with sexine (1–1.2 µm, visible columellae max. 0.3 µm long) thicker than nexine 0.3–0.5 µm).

#### *R. microcarpum* K.M Feng ex. T.L. Ming (Figs. 2D, D1, 3D)

Pollen grains tricolporate, suboblate to subprolate, triangular to circular in polar view and angular, elliptical to subcircular in equatorial view (Figs. 1, 2); measurements under LM: polar axes 31.8–32.7 µm, equatorial axes 37.3–42.4 µm; under SEM: polar axes 26.2 to 28.8 µm, equatorial axes 26.6–31.2 µm; endoporus rectangular to quadrangular 10.4–11.9 µm × 5.9–9.1 µm. Tectum: tectate, perforate, fossulate, micro-verrucate to micro-rugulate, mesocolpium areas more pronounced perforate and micro-verrucate and colpus margins and polar areas more micro-rugulate and fossulate; micro-verrucae and micro-rugulae regularly covered by supratectal micro-echini (or micro-gemmae), occasionally arranged in rows and fused (the micro-rugulae considerably smaller than micro-rugulae of *R.* *macrocarpum*); colpus membrane when visible loosely micro-verrucate; pollen wall thickness 1.0–1.3 µm with sexine (0.7–0.9 µm, visible columellae max. 0.2 µm long) thicker than nexine (0.3–0.4 µm).

#### *R. indochinense* H.L. Li (Figs. 2E, E1, 3E)

Pollen grains tricolporate, subspheroidal to suboblate or subprolate, triangular to circular in polar view and angular, elliptical to subcircular in equatorial view (Figs. 1, 2); measurements under LM: polar axes 26.9–29.1 µm, equatorial axis 30.1–38.2 µm; and under SEM: polar axes 23.5–27.1 µm, equatorial axes 23.1–37.5 µm; endoporus shape rectangular to quadrangular, 5.4–9.1 × 5.4–6.7 µm. Tectum: tectate, perforate, rugulate, shallow fossulate, rugulae generally angular, irregularly shaped and bordered by shallow fossulae (grooves) and perforations; rugulae 1–2 µm long, generally < 1 µm wide, regularly ornamented with supratectal blunt micro-echini (or micro-gemmae), decreasing considerably in size towards colpus margins; perforations most prominent in polar areas, diminishing towards colpus margins; colpus membrane micro-verrucate; pollen wall thickness 1.4–1.5 µm with sexine (0.8–1 µm, visible columellae max. 0.3 µm long) thicker than nexine (ca. 0.4 µm).

#### *R. truongsonense* P.W. Fritsch, W.B. Liao & W.Y. Zhao (Figs. 2F, F1, 3F)

Pollen grains tricolporate, suboblate to spheroidal, triangular to circular in polar view and angular, elliptical to subcircular in equatorial view (Figs. 1, 2); measurements under LM: polar axes 30.5–32.7 µm, equatorial axes 36.4–40.0 µm; and under SEM: polar axes 26.1–28.2 µm, equatorial axes 28.3–32.1 µm; endoporus rectangular 6.3–12.7 µm × 4.3–7.4 µm. Tectum: tectate, covered with regularly arranged micro-gemmae and fused micro-gemmae producing short, rod-like structures, perforate to foveolate, tectum becoming more pronounced micro-rugulate to micro-verrucate and fossulate towards colpus margins; colpus membrane, when visible loosely micro-verrucate; pollen wall thickness 1.0–1.4 µm with sexine (0.8–0.9 µm, visible columellae max. 0.3 µm long) thicker than nexine (ca. 0.5 µm).

### Description of fossil pollen

#### *Rehderodendron* sp. 1 (Figs. 3G, 4A).

**Fig. 4:**
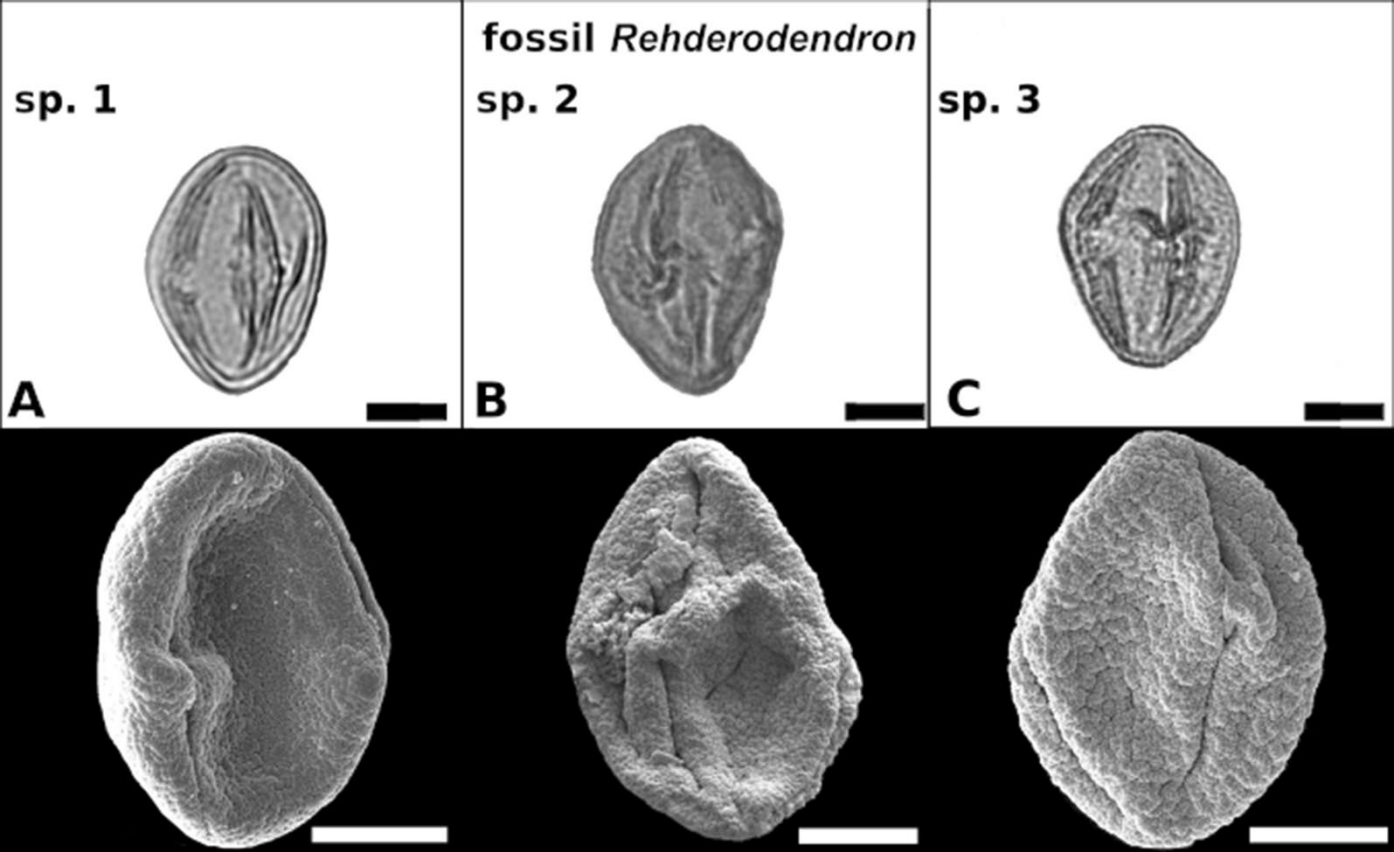
LM and SEM overview images of three examples of fossil *Rehderodendron* pollen. *R*. sp. 1 from the lower Eocene of England, *R*. sp. 2 from the middle Miocene in Austria (Schaßbach), *R.* sp. 3 from the middle Miocene in German, (Entrischenbrunn)

Pollen grains tricolporate, prolate; polar axis ca. 30.5 µm and equatorial axis ca. 22.3 µm (SEM); endoporus ± quadrangular to rectangular, ca. 7.2 µm × ca. 5.7 µm (compressed fossilized state). Tectum: rugulate, fossulate, perforate, covered with supratectal micro-gemmae, the rugulae ± angular, occasionally curving 0.6–1.7 µm long and 0.4–0.8 µm wide; micro-gemmae regularly arranged in rows perpendicular to the rugulae lengths; wall thickness 1.2–1.4 µm with sexine thicker than nexine (Figs. 2, 3).

Remarks: This pollen type comes from the PETM section recovered in exploration drill cores of the London tube in Brixton (England). It was originally affiliated with Ebenaceae (*Diospyros* in [22]) but clearly is *Rehderodendron*. It most closely resembles *R.* *kwangtungense* and *R.* *macrocarpum*.

#### *Rehderodendron* sp. 2 (Figs. 3H, 4B)

Pollen grains tricolporate, prolate; polar axis 30.8–34 µm and equatorial axis 23.2 –24.4 µm; endoporus more-or-less quadrangular to rectangular, 3.8–4.9 × 3.8–4.6 µm (compressed fossilized state). Tectum: irregularly shaped, rugulate to micro-rugulate to irregularly shaped verrucate, fossulate, perforate, rugulae ± angular, occasionally curving (0.7–2.5 × 0.2–0.8 µm) and ornamented with striae or linearly fused supratectal micro-gemmae arranged perpendicular to the length of the rugulae; wall thickness 1.2–1.3 µm with sexine thicker than nexine (Figs. 2, 3).

Remarks: This pollen type has been reported [29 Fig. 3G–I] from the middle Miocene Schaßbach clay pit (Austria; MMTM). It resembles a mixture of *R.* *kwangtungense,* *R.* *microcarpum* and *R.* *indochinense*.

#### *Rehderodendron* sp. 3 (Figs. 3I, 4C)

Pollen grains tricolporate, prolate; polar axis 31–34.6 µm and equatorial axis 23.2–24.5 µm; endoporus more-or-less quadrangular to rectangular ca. 4.8–6.7 µm × 4.1–5.6 µm (compressed fossilized state). Tectum: rugulate, fossulate, perforate, rugulae are more-or-less angular, occasionally curving ca. 0.8–1.8 µm long and ca. 0.3–0.8 µm wide and ornamented with striae arranged diagonally or perpendicular to the length of the rugulae; wall thickness ca. 1.1–1.3 µm with sexine thicker than nexine (Figs. 2, 3).

Remarks: This pollen type has been reported from Hofmann and Sachse [31] middle Miocene (end of the MMTM) sand pit in Entrischenbrunn (Germany). It most closely resembles a mixture of *R.* *macrocarpum,* *R.* *microcarpum* and *R.* *indochinense*.

### Summarized results of pollen descriptions

The sexine sculpture and ornamentation of *R.* *macrocarpum* and *R.* *microcarpum* exhibit fluent continuous transitions in the rugulae sizes (larger to smaller); however, *R.* *macrocarpum* is more fossulate and less perforate whereas *R.* *microcarpum* displays more perforations (Fig. 3). In comparing the sexine of *R.* *microcarpum* with *R.* *kwangtungense* and *R.* *indochinense* the rugulae sizes are also transitional (towards smaller and less pronounced rugulae and more obvious perforations); however *R.* *kwangtungense* has more pronounced supratectal micro-gemmae (Fig. 3). Separation among these four taxa is indistinct. Conversely, *R.* *truongsonense,* which is conspicuously perforate with supratectal micro-gemmae or echini, and *R.* *kweichowense,* which is micro-verrucate to areolate (Fig. 2), can be easily differentiated from each other and the rest of the extant species, and the fossil pollen, which are neither conspicuously perforate and micro-gemmate, nor micro-verrucate to areolate.

### Brief descriptions of extant fruits

**Fig. 5:**
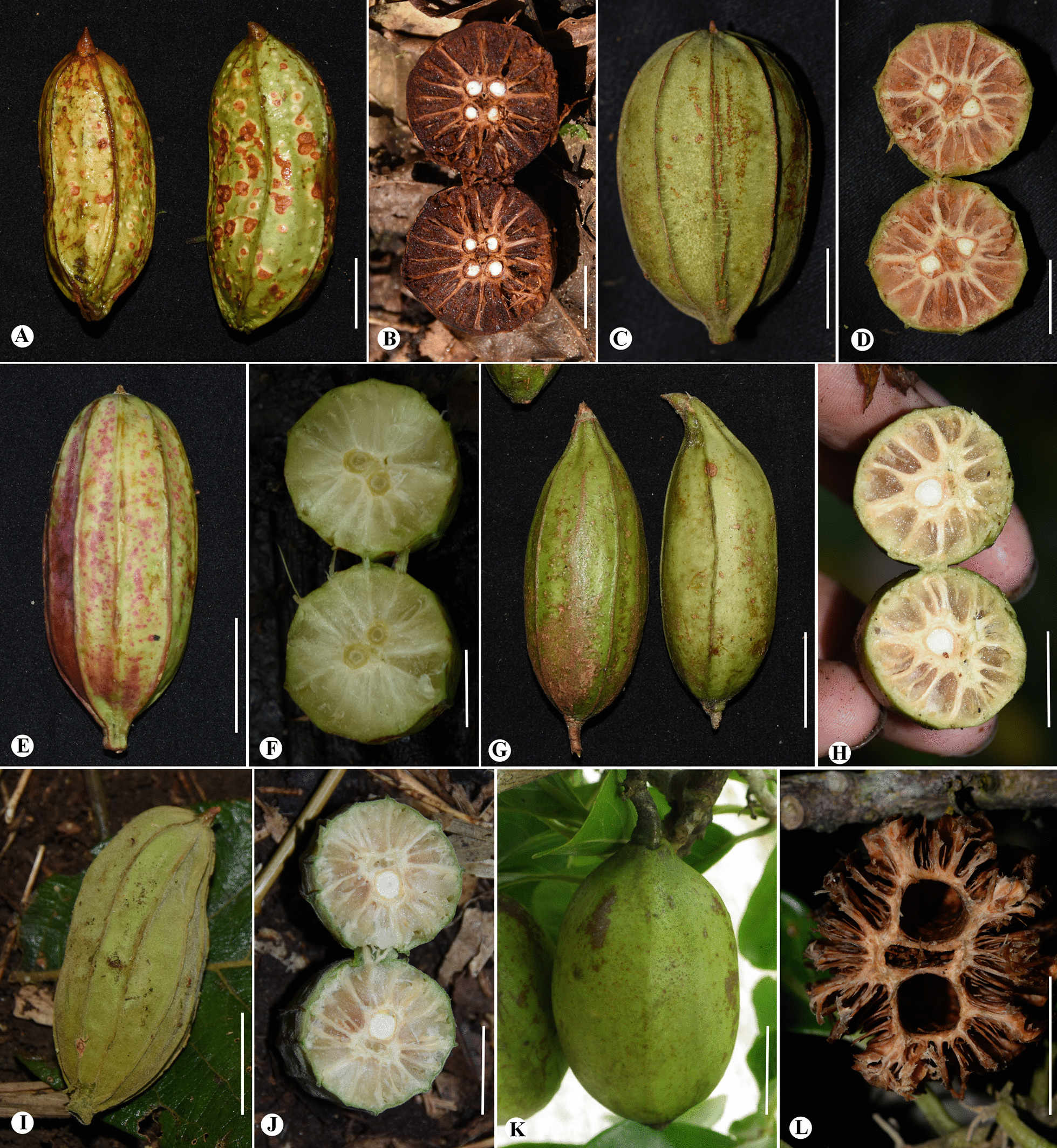
Images of the six extant *Rehderodendron* fruits: **A**, **B** *R.* *indochinense*; **C**, **D** *R.* *kwangtungense*; **E**, **F** *R.* *macrocarpum*; **G**, **H** *R.* *microcarpum;* **I**, **J** *R.* *kweichowense;* **K**, **L** *R.* *truongsonense.* *S*cale bar 2 cm

 In general all investigated species develop a thick spongy mesocarp and differ mostly in the rib number and complexity of the endocarp ray system (Fig. 5, Table 3).

**Table 3:** Fossil occurrences of fossil Rehderodendron fruits (f) and pollen (p) in Europe

Fossil taxon	Organ	Age	Country	Locality	Authors
*R.* *stonei*	f	Lower Eocene	England	London Clay	Mai, 1970
*R* *sp.*	p	Lower Eocene	England	Brixton drill core	Hofmann, 2018 (as *Diospyros*)
*R.* *stonei*	f	Lower/middle Eocene	France	Sabal D’Anjou	Vaudois-Mieja 1983
*R.* *sp.*	p	Oligocene/Miocene	Austria	Hinzenbach	Filek, 2019
*R.* *ehrenbergii*	f	Lower/upper Miocene	Germany	Düren, Wiesa	Mai, 1970
*R* *sp.*	p	Early Miocene	Austria	Köflach-Voitsberg	Kovar-Eder et al., 1998
*R.* *sp.*	p	Early Miocene	Austria	Korneuburg	Hofmann et al., 2002
*R.* *wiesaense*	f	Early Miocene	Germany	Wiesa	Mai, 1970
*R.* *wiesaense*	f	Early Miocene	Germany	Wiesa	Geisert and Gregor, 1981
*R* *sp.*	p	Early Miocene	Germany	Wiesa	Vomela, 2016
*R.* *ehrenbergii*	f	Miocene	Germany	Oberpfalz	Gregor, 1978
*R.* *custodum*	f	Middle Miocene	Czech R	Zittau	Holý, 1975
*R* *sp.*	p	Middle Miocene	Germany	Kreuzau	Ferguson et al., 1998
*R* *sp.*	p	Middle Miocene	Germany	Randeck Maar	Kottik, 2002
*R* *sp.*	p	Middle Miocene	Austria	Lavanttal	Grimsson et al., 2020
*R* *sp.*	p	Middle Miocene	Austria	Schasbach	Hofmann & Lichtenwagner, 2020
*R* *sp.*	p	Middle Miocene	Germany	Entrischenbrunn	Hofmann & Sachse, 2023
*R.* *ehrenbergii*	f	Miocene/Pliocene	France	Elsass	Geissert et al., 1990
*R.* *ehrenbergii*	f	Lower Pliocene	Italy	several localities	Martinetto, 1998
*R.* *dacicum*	f	Late Pliocene	Romania	Racosu-de-Sus	Mai & Petrescu, 1983

The fruits of *R.* *indochinense* (Fig. 5A, B) have a characteristic long cylindrical shape with five obvious ribs, and the fruit surface usually has large brown spots, a unique feature among extant *Rehderodendron* species. The styles are persistent, the stigma is beaked, and the endocarp rays are irregular. 

*Rehderodendron* *kwangtungense* (Fig. 5C, D) generally is characterized by columnar fruits that are conspicuously ribbed, and the styles are inconspicuous and persistent. The endocarp ray system is complex and displays irregular rays.

The fruits of *R.* *microcarpum* (Fig. 5E, F) are more narrower and smaller than all other *Rehderodendron* fruits with usually an ovoid, cylindrical to fusiform shape and an inconspicuously ribbed surface (5 ribs usually visible). The styles are persistent (conical coracoid). The endocarp has simple, thickened rays. 

*Rehderodendron* *macrocarpum* (Fig. 5G, H) is characterized by its oblong to elliptic fruits that are conspicuously ribbed (8–12 ribs); persistent styles are very short. The endocarp rays are thick and its rays display irregular thicknesses and lengths.

As mentioned above, the pollen morphology of *R.* *kweichowense* and *R.* *truongsonense* differ substantially from those of the other four species investigated. This difference is also reflected in their fruits: the fruits of *R.* *kweichowense* (Fig. 5I, J) are densely covered with stellate hairs. The fruits also have 10 to12 ribs and irregular endocarp rays. The fruits of *R.* *truongsonense* (Fig. 5K, L) are short-terete and inconspicuously ribbed; the endocarp is thickened and comprises an even more complex endocarp ray system than *R.* *macrocarpum*.

## Discussion

### Comparison of extant and fossil pollen of *Rehderodendron*

The comparison of LM and SEM images of the three fossil and six extant *Rehderodendron* pollen described here revealed that the fossil pollen resemble overall four extant *Rehderodendron* taxa: the lower Eocene *Rehderodendron* sp. 1 from the PETM (Figs. 3G, 4A) resembles a mixture of mostly *R.* *kwangtungense* and *R.* *macrocarpum* (Figs. 2A,C, 3A, C), the middle Miocene *Rehderodendron* sp. 2 from the MMTM (Figs. 3H, 4B) resembles a mixture of *R.* *kwangtungense*, *R.* *microcarpum*, and *R.* *indochinense* (Figs. 2A, D, E, 3A, D, E), and sp. 3 from the end of the MMTM (Figs. 3I, 4C) resembles a mixture of *R.* *macrocarpum*, *R.* *indochinense* and *R.* *microcarpum* (Figs. 2C, E, D, 3C, E, D). However, there are two differences:

1. The fossil pollen are always prolate (Fig. 3A–C), which is only the case in extant *Rehderodendron* pollen when acetolysed for a longer time. The influence of acetolysation under heat is therefore assumed to mimic the fossilization process and the pollen shape can change from suboblate to prolate (Fig. 2A1–F1).
2. As compared to the extant species *R.* *indochinense,* *R.* *kwangtungense,* *R.* *macrocarpum* and *R.* *microcarpum,* the rugulae of the fossil *Rehderodendron* sp. 2 and sp. 3 display a much wider variation in size, and the supratectal arrangement of micro-gemmae on the rugulae in the fossil specimens of *Rehderodendron* sp. 2 and sp. 3 is generally more diagonally arranged and the individual micro-echini/micro-gemmae are mostly fused into rows (Figs. 2, 3).

However, rugulae size and the degree of fusion of supratectal echini is also various within extant and fossil species. Both, extant and fossil pollen grains show a decrease in rugulae size towards the colpus margo (Fig. 3). There are more than those above: fossil pollen grains were found in Austria and Germany and are of late Oligocene to middle Miocene age [30, 34–39] (summary Table 3). They all appear similar to *Rehderodendron* species sp. 2 and sp. 3 described here and display the same variation of rugulae size and fusion of supratectal micro-gemmae. The resemblance of fossil pollen and fruits to *R.* *indochinense* begins in the Miocene. We suggest that all fossils were members of a lineage leading to the extant species.

No records of fossil *Rehderodendron* pollen resembling the extant taxa *R.* *kweichowense* and *R.* *truongsonense* exist. Our assessment of the fossil *Rehderodendron* pollen type in Grímsson et al. [30: Fig. 20D-F] affiliated with *R.* *kweichowense* and *R.* *macrocarpum* was hampered by a mix-up of taxa and insufficient images in [23]: their *R.* *macrocarpum* is actually *R.* *indochinense* and our new images show that *R.* *kweichowense* has a completely different tectum ornamentation than the fossil pollen in [30]. Consequently, similar to the other fossil pollen taxa, their pollen taxon resembles a mixture of *R.* *indochinense* and *R.* *microcarpum.*

### Distribution of fossil *Rehderodendron* fruits and comparison with extant species

Manchester et al. [3, 18, 26–28] provided a brief summary of *Rehderodendron* fossil diaspores. There are additional fossil diaspore occurrences in the literature [e.g., 20, 25, 40–42] that are listed in Table 4. Five fossil taxa have been recorded from Europe [3, 18, 27]: *R.* *stonei* (Reid & Chandler) Mai from the lower Eocene to mid-Eocene of England [19] and France (near the EECO, *R.* *ehrenbergii* (Kirchheimer) Mai, *R.* *wisaense* Mai and *R.* *custodum* Holý from the Miocene in Germany and the Czech Republic (often near the MMTM), *R.* *ehrenbergii* from the Pliocene in Italy, and *R.* *dacium* Mai & Petrescu from the Pliocene in Romania. According to Manchester et al. [3], who reinterpreted the findings of Miki [43], *Rehderodendron* diaspores probably also were present during the Pliocene in Japan, but this material has not been re-investigated. The fossil fruits have generally been compared with *R.* *kwangtungense* (= *R.* *hui* in [18 page 489; 24 page 67]), perhaps because the material of of *R.* *kwangtungense* was the easiest to access and the only one available in European herbaria.

**Table 4:** Measurements and descriptions of fossil and extant *Rehderodendron* fruits

Fruit taxon	Length mm	Width mm	Discus	Sepals	Lacunae	Ribs/Rays	Shape	Preservation	Authors	Interpretation this paper
*R.* *stonei*	16–30	6.5–22	Short and wide	Persistent	2–3, wide lumen	9	Small egg-shaped	Pyritic preservation	Mai, 1970 Manchester et al. 2009	*R.* *kwantungense* *R.* *microcarpum*
*R.* *ehrenbergii*	24 (33) 45	13.5–30	Medium clavate	None	(1) 2 (3), wide lumen	9–11?	Egg- to broad spindle-shaped	Carbonaceous	Mai, 1970 Manchester et al. 2009	*R.* *kwantungense* *R.* *macrocarpum* *R.* *microcarpum*
*R.* *wiesaense*	55	> 21	Big and pointed	Vascular bundles	2, narrow lumen	10?	Broad spindle-shaped	Steinkern	Mai, 1970	*R.* *indochinense*
*R.* *dacicum*	18 (27) 41	7 (12) 17	Blunt and short	None	1?	5 (7) 8	Elongate egg- to spindle-shaped	Carbonate perminerali sation	Mai & Petrescu 1983	cf. *R.* *indochinense*
*R.* *custodum*	17 (23) 36	8 (10) 15	Promi-nent	None	1–2 narrow lumen	> 8	elongate egg- to slim spindle shaped	?	Holý, 1975	cf. *R.* *indochinense*
*R.* *kweichowense*	45–75	30–45				10–12	Long cylindrical	Fresh	This paper	
*R.* *kwangtungense*	45–80	25–40				10–12	Oblong, obovate or elliptic	Fresh	This paper	
*R.* *macrocarpum*	35–90	25–35		Persistent		10–12	Oblong, obovate or elliptic	Fresh	This paper	
*R.* *microcarpum*	48–75	20–29				5 obvious, others unconspi-cuous	Fusiform, ovoid to columnar	Fresh	This paper	
*R.* *truongsonense*	35–70	25–40				Unconspi-cuous	Cylindrical, elliptic	Fresh	This paper	
*R.* *indochinense*	45–110	28–60		Unconspi-cuous		5–10	Long cylindrical	Fresh	This paper	

In comparing fruit morphology of fossil *Rehderodendron* with extant species it can be seen that the fossil fruits do not resemble only *R.* *kwangtungense* (Fig. 5C, D) as suggested by Mai [18] and Gregor [24], but other species as well*.* The smallest fruit is that of *R.* *stonei* [3, Figs. 50–51 and in 18, Fig. 17j-l], displaying as well characters of *R.* *microcarpum* (Fig. 5G, H), whereas the medium-sized *R.* *ehrenbergii* [e.g., 3, Figs. 45–49; 18, Fig. 17 g-h] more closely resembles *R.* *macrocarpum* (Fig. 5E, F), and *R.* *microcarpum* (Fig. 5G, H). The largest fruit is that of *R.* *wisaense* [18, Fig. 17 m, plate 69 Figs. 15–17] and resembles a poorly developed fruit of *R.* *indochinense* (Fig. 5A, B); the same is most likely true for *R.* *dacium* and *R.* *custodum* (Table 3). We conclude that, like the pollen data, the fossil fruits display the same variation and mixture of morphological characteristics as in extant *R.* *kwangtungense*, *R.* *microcarpum*, *R.* *macrocarpum*, and *R.* *indochinense*, and the resemblance to *R.* *indochinense* occurs in fossil fruits from the Miocene to Pliocene.

### Eurasian distribution of extant and fossil *Rehderodendron*

**Fig. 6:**
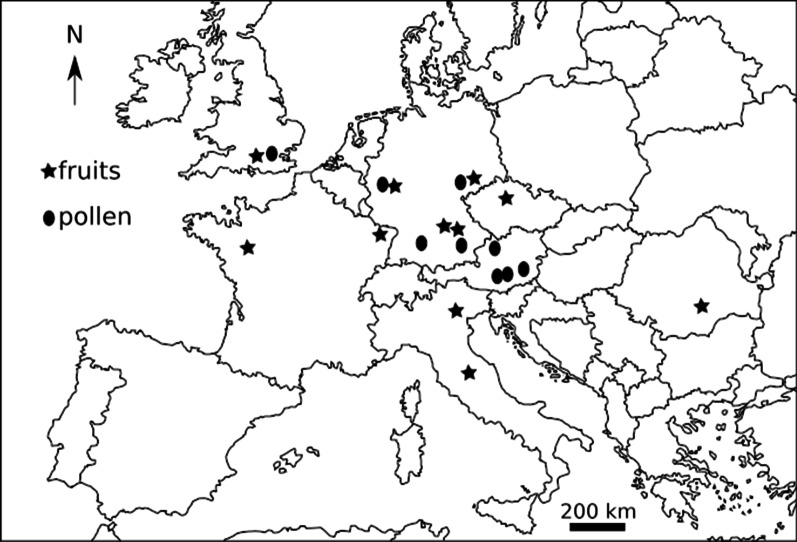
Map of Europe showing geographic distribution of *Rehderodendron* fossils in Table 3

The geographic distribution of extant *Rehderodendron* and its fossil fruits and pollen shows a disjunction up to the Late Miocene (or Pliocene):

1. The extant species occur solely in Asia (mainly China, Vietnam and Myanmar, Laos, NE India; Tables 1, 5, Fig. 1) and grow generally in Cwa, Cwb, Cfa climates of the Köppen-Geiger classification ([44, 45]: warm-temperate climate with dry winters and hot or warm summers or fully humid with hot summers) or Cw and Cf climates of the Köppen-Trewartha classification ([46]: subtropical climate with dry winters or fully humid). The exception is the Vietnamese *R.* *truongsonense* [15], which thrives as well under Am climate of the Köppen-Geiger classification ([44, 45]: tropical to subtropical monsoon climate). Fossils similar to *R.* *truongsonense* and *R.* *kweichowense* are not represented in the fossil record and therefore will be not discussed further. In comparing temperature and rainfall ranges extracted from the climate information based on extant *Rehderodendron* distribution from WorldClim website (http://worldclim.org/version2; [47], see Table 5), many of the modern species of *Rehderodendron* appear to easily adapt to subtropical monsoon climate (the annual mean temperature of *Rehderodendron* ranges from 7.08–19.5 ℃ and annual precipitation range from 893–3856 mm; Table 5).
2. The fossil occurrences of pollen and fruits that all resemble extant *R.* *kwangtungense*, *R.* *macrocarpum*, *R.* *microcarpum,* and *R.* *indochinense* are restricted to Europe (England, France, Czech Republic, Germany, Austria, Italy and Romania; Table 4, Fig. 6) and until now have not been found in other regions. *Rehderodendron* fossils are often recorded from exceptionally warm periods during the early Eocene around the PETM in England [48–51, 51] and EECO in France [20], these are periods characterized by A and C climates of Köppen-Geiger [44, 45; warm temperate to tropical climate] and C climates of Köppen-Trewartha classification [46; subtropical climate]. Further, fossil pollen occur at the Oligocene/Miocene transition, during the lower and middle Miocene (MMTM), periods that are characterized by mostly C and less A climates of Köppen-Geiger (Köppen-Geiger data of the MMTM in Germany Entrischenbrunn [31], Köppen-Geiger data of the MMTM in Austria Lavanttal [30], and CLAMP and Köppen-Geiger data of Schaßbach, Austria [52, 53]). Fossil fruits occurred at the Miocene/Pliocene transition and during the Pliocene in southern Europe in refugial “warm and moist niches” [54, 42 for Italy; 27 for Romania]. The palaeoclimate conditions can be summarized as sufficient warmth [42, 54] and either evenly distributed precipitation/humidity (Cfa climate), or unevenly distributed precipitation during the year (= Cwa/Cwb climate = drier winter and warm or hot summers) to grow, flower, set seed and propagate. During the Pliocene, the early lineage of *Rehderodendron* went extinct in Europe because of cooling and drying during the Pleistocene [6, 7]. Consequently, the European early *Rehderodendron* was likely an ancestral lineage leading to the extant species (*R.* *indochinense*, *R.* *kwangtungense*, *Rehderodendron* *macrocarpum* and *R.* *microcarpum*) that currently occur in China, Myanmar and Vietnam, whereas *R.* *kweichowense* and *R.* *truongsonense* likely evolved separately.

**Table 5:** Temperature and rainfall ranges of extant *Rehderodendron* species extracted from WorldClim website (http://worldclim.org/version2; [47])

*Rehderodendron* species	Annual mean temperature C°	Mean C°	Annual precipitation in mm	Mean in mm	Precipitation of wettest month in mm	Mean in mm	Precipitation of driest month in mm	Mean in mm
*R.* *macrocarpum*	7.08–14.58	10.61	893–2105	1339.6	136–524	272.36	9–41	21.63
*R.* *kweichowense*	11.00–18.08	14.01	1393–2427	1815.2	216–534	343.29	22–45	31.21
*R.* *kwangtungense*	9.08–17.58	13.02	1197–2803	1984.4	190–516	346.21	26–68	46.68
*R.* *indochinense*	11–16.83	13.42	1435–2930	1996.3	298–582	403.82	21–36	12
*R.* *truongsonense*	12.08–17.00	14.38	1859–3856	2769.8	365–990	584.75	25–51	38
*R.* *microcarpum*	9.42–17.17	14.2	1294–4336	2020.8	201–955	405.45	13–28	16.55

### The dispersal from Europe to Asia

Fruit dispersal of several taxa by animals and water and the west–east migration during the Eocene from Europe to China and vice versa has been mentioned, amongst other fossil taxa, for *Juglans* Linnaeus of section *Cardiocaryon*, *Cornus* Linnaeus of the blue and white fruited clade, *Nyssa* *sinensis* Olivier type, and *Symplocos* Jacquin subgenus *Palura* from middle Eocene strata of Hainan [33]. Additionally, a “boreotropical” origin of the entire family of Juglandaceae has been implied by [55], who suggested that Europe played a critical role in the migration and distribution of taxa but also exhibited high extinction of Juglandaceae taxa [55, Fig. 4]. Fitting also into this scheme is *Symplocos,* with its well known fossil record in Europe [summarized in 56] ranging from Paleogene to late Pliocene. The relationship of European *Symplocos* fossils with Asian *Symplocos* taxa and a Eurasian origin for the Symplocaceae has been demonstrated by Manchester and Fritsch [57, 58]. The family was known also from a few Eocene and Miocene localities in North America and now has a disjunct east Asian-American distribution [59, 60]. There are more examples of taxa with a “circumboreal connection” [2, Fig. 2] which were distributed from the Paleogene onwards up to Miocene or Pliocene in Eurasia and North America and nowadays are present in east Asia only: examples are *Torricellia* De Candolle [58, 61–63], *Gordonia* J. Ellis (= *Polyspora* Sweet [24, 29, 63–65]), and Mastixiaceae [e.g., 1, 19, 59, 66–68]. Our data from *Rehderodendron* are consistent with the idea that this European-Asian connection must have been still active during the Miocene across the then-closed Turgai Strait [1, 18, 42, 57, 69].

The modern species of *Rehderodendron* generally grow along streams and stream valley slopes (such as *R.* *kweichowense*, *R.* *macrophyllum*, *R.* *microcarpum, R.* *truongsonense*), or on the gentle slopes in mountain cloud forests (*R.* *indochinense,* *R.* *kwangtungense, R.* *macrocarpum*). This indicates that water and gravity are important propagation forces for the fruits of this genus and that their thick spongy mesocarp helps them to float in water. Based on field observations, fruits of *Rehderodendron* should not float in water for a long time, or else they will rot. Additionally, it has been observed that some rodents (such as squirrels) collect fruits of *Rehderodendron* for consumption and storage (dyszoochory behaviour), but often destroy the seeds in the fruits and therefore only a small fraction of seeds might be able to germinate in the (forgotten) storage. We therefore suggest that water (and gravity) are the main driving vectors for the lateral fruit distribution and migration of *Rehderodendron* downslope whereas animals might be responsible for the fruit distribution within the mountain areas.

The dispersal of *Rehderodendron* to the east may have occurred in three stages.

The existence of a continuous zone of maritime-influenced vegetation along the Tethys during the Cenozoic and the concept of a “boreotropical flora” proposed by Wolfe [5, see also 1, 70] may have played a role in the early dispersal events of *Rehderodendron* species and many other taxa characteristic of the Eurasian relict flora (see above) (1.) The lower Eocene pollen and fruits from England and the Miocene fossils from Germany and Austria all show similarities, amongst others, with *R.* *kwangtungense*. *Rehderodendron* *kwangtungense* could be interpreted to represent the oldest developed extant species (Zhao, unpublished data) and might have dispersed eastwards already during middle Eocene times; it therefore can be found today in the easternmost part of China (see Fig. 1).

(2.) The ongoing Tethys closure and subsequent uplift of the Tibetan Plateau likely hampered the dispersal of *Rehderodendron* between Europe and East Asia. Additionally, the disappearing Tethys must have shifted the former Eurasian maritime-influenced climate to a more continental monsoonal climate [6, Fig. 2; 7], resulting in the evolution from the Eocene *Rehderodendron* *stonei* and *Rehderodendron* pollen sp. 1 to *Rehderodendron* *ehrenbergii* and *R.* *wisaense* etc*.* and to *Rehderodendron* pollen sp. 2 and sp. 3 during the Miocene (these taxa resemble in part *R.* *indochinense*) (3.) The cooling during the Pliocene [6 Fig. 2; 7] caused the extirpation of European *Rehderodendron* (the extant species of *Rehderodendron* require annual mean temperatures > 7.08 ℃, and annual precipitation > 893 mm; Table 5). Although the fruits of *Rehderodendron* are highly variable, it is apparent that fruits of *R.* *ehrenbergii* are similar to extant fruits of *R.* *microcarpum*, which mostly is distributed in the western part of the distribution of the range of the genus (southern eastern Himalaya; Yunnan and Xizang in China, and Kachin in Myanmar, Fig. 1). If so, then this refugium represents the end of the eastward migration of *R.* *ehrenbergii*, however, there is no evidence that the fossil *R.* *ehrenbergii* and the extant *R.* *microcarpum* belong to the same species. Never-the-less, the southeastern Himalaya was warmer than southeastern Europe during the Pleistocene ice age, which could explain why *R.* *microcarpum* survived while *R.* *ehrenbergii* perished in Europe. A comparable migration route can be assumed for the Miocene and Pliocene *R.* *wiesaense*, *R.* *dacium,* and *R.* *custodum* the fruits of which resemble mostly the extant *R.* *indochinense*, which is distributed slightly farther south of *R.* *microcarpum* (Fig. 1).

A somewhat comparable fossil (Eocene and Miocene) and modern distribution pattern to *Rehderodendron* can be found in *Torricellia* De Candolle (Torricelliaceae (Wangenheim) H.H. Hu) [2, 61–63]: one extant species (*T.* *tiliifolia* De Candolle) lives in the eastern Himalaya and the other one (*T.* *angulata* Oliver) in central China [63 page 317].

## Conclusions

Fossil pollen grains of *Rehderodendron* occurred in Europe from the lower Eocene to Miocene and resembles a mixture of characters of the extant *R.* *indochinense,* *R.* *kwangtungense,* *R.* *macrocarpum*, and *R.* *microcarpum*. Fossil fruit of *Rehderodendron* occurred only in Europe from the lower Eocene to the Pliocene (southern Europe) and resemble a mixture of characters of the same species. Fossil pollen grains and fruits with the morphology of *R.* *kweichowense* and *R.* *truongsonense* are not known and appear to represent a different lineage within *Rehderodendron*.

Fossil *Rehderodendron* in Europe grew under warm-temperate to subtropical climate conditions, generally A and C climates of Köppen-Geiger, which is also true for the extant species (China, Vietnam and Myanmar). Fossil *Rehderodendron* was frequently found in warm and humid periods of the Cenozoic (PETM, EECO, MMTM) and the last warm moist periods of the Pliocene in southern Europe.

Today *Rehderodendron* is suggested to be an element of the Eurasian “boreotropical” relictual flora which dispersed from Europe to Asia along the Tethys Sea (most probably via water) from the middle Eocene onwards and became extinct in Europe after the Pliocene.

## Data Availability

All specimens are available at the listed repositories: stubs with pollen at the University of Vienna, Department of Palaeontology and herbarium specimens at Sun Yat-sen University State Key Laboratory and Guangdong Key Laboratory of Plant Resources.
